# Scoping Review of Predictors and Risk Factors for Acute Myocardial Infarction in Patients with Chronic Kidney Disease: Short title: Acute Myocardial Infarction in Patients with CKD

**DOI:** 10.31661/gmj.v15i.4090

**Published:** 2026-02-02

**Authors:** Roohollah Rahbani, Farid Taghavi, Mohsen Abbasnezhad, Mehdi Maleki, Leila Liaghat, Hamid Ghazipoor

**Affiliations:** ^1^ I.M. Sechenov First Moscow State Medical University, Moscow, Russia; ^2^ Cardiovascular Research Center, Tabriz University of Medical Sciences, Tabriz, Iran; ^3^ Department of Family Medicine, School of Medicine, Shiraz University of Medical Sciences, Shiraz, Iran

**Keywords:** Chronic Kidney Disease, Myocardial Infarction, Predictors, Biomarkers, Risk Scores, Dyslipidemia, Inflammation

## Abstract

**Background:**

Chronic kidney disease (CKD) significantly elevates the risk of acute myocardial infarction (AMI) and associated mortality, with cardiovascular disease accounting for approximately 40% of deaths in CKD patients. Diagnostic and therapeutic challenges arise due to altered biomarker profiles and underutilization of interventions like percutaneous coronary intervention (PCI). This systematic review aims to identify and evaluate predictors of AMI in CKD patients, focusing on clinical, biochemical, demographic, and lifestyle-related risk factors to inform targeted prevention strategies.

**Materials and Methods:**

Following PRISMA guidelines, a systematic search was conducted across PubMed, Embase, Scopus, Web of Science, and Cochrane Library up to August 14, 2025, using MeSH terms like “chronic kidney disease” and “myocardial infarction.” Included studies involved adult CKD patients, assessing predictors through observational designs or relevant randomized controlled trial subgroup analyses. Data were extracted by two independent reviewers, with study quality assessed using the Newcastle-Ottawa Scale. Narrative synthesis was employed due to heterogeneity.

**Results:**

Twenty studies (54–911,360 participants) identified dyslipidemia, inflammatory (CRP, IL-6) and cardiac biomarkers (NT-proBNP, hs-cTnT), lower eGFR, albuminuria, advanced CKD stage, hypertension, prior AMI, and older age as key AMI predictors. Risk scores (GRACE, PRECISE-DAPT) and novel indices (TyG, TyG-BMI) showed high predictive accuracy.

**Conclusion:**

Comprehensive risk assessment integrating biomarkers, clinical factors, and novel indices can enhance AMI prediction and guide interventions in CKD patients, addressing therapeutic underutilization.

## Introduction

CKD significantly increases the incidence of AMI, with a rate of 2.5 cases per 1000 people per year in CKD stages G3–G5 [1]. Patients with CKD face a higher risk of short- and long-term mortality following non-ST-segment-elevation myocardial infarction, with hazard ratios of 1.24 at 30 days, 1.47 at 1 year, and 1.55 at 5 years compared to non-CKD patients [2]. Cardiovascular disease accounts for approximately 40% of deaths in CKD patients, with sudden cardiac death comprising up to 60% in dialysis patients [3].

The diagnostic pathway for acute myocardial infarction (AMI) in patients with chronic kidney disease (CKD) differs significantly from that in non-CKD patients due to altered biomarker profiles. Chronically elevated high-sensitivity cardiac troponin (hs-cTn) in CKD reduces the diagnostic specificity for AMI [4]. Percutaneous coronary intervention (PCI) is underutilized in this population despite its ability to reduce mortality across CKD stages [5]. Lower utilization of aspirin and reperfusion therapies in severe CKD contributes to higher mortality rates [6]. Preventive measures, such as statin therapy, are underutilized in CKD patients despite reducing cardiovascular events by 22% in non-dialysis-dependent patients [7]. Patients with CKD are less likely to receive P2Y12 inhibitors, invasive angiography, percutaneous coronary intervention, or cardiac rehabilitation, contributing to worse outcomes [2]. In acute settings, peak hs-cTnI outperforms hs-cTnT for diagnosing coronary artery disease in CKD [8]. Serial hs-cTnI measurements improve non-ST-elevation AMI diagnosis in CKD [9]. Serial hs-cTn changes, combined with risk scores and adjusted biomarkers, could guide timely interventions like PCI to address therapeutic underutilization and reduce mortality in CKD patients [4, 5].

Risk factors for AMI in CKD include advanced CKD stage, older age, male sex, hypertension, and low educational level, while prior AMI significantly increases the risk of subsequent events [1]. Hemodialysis exacerbates AMI risk through immune dysfunction, lipid metabolism disorders, and vascular calcification, necessitating targeted prevention strategies [10]. Statin treatment is recommended for CKD patients over 50 with estimated glomerular filtration rates below 60 mL/min/1.73m² to mitigate coronary risk [7]. In case of prevention and prediction of risk of MI in CKD, while many studies are performed in this era, there isn’t any comprehensive reviewing paper for this.

## Methodology

This systematic review was performed in adherence to the Preferred Reporting Items for Systematic Reviews and Meta-Analyses (PRISMA) guidelines on electronic databases, including PubMed/MEDLINE, Embase, Scopus, Web of Science, and the Cochrane Library, from inception up to August 14th 2025. Only studies published in English were included. Gray literature sources were not retrieved.

The search strategy combined Medical Subject Headings (MeSH) terms like”chronic kidney disease,” “CKD,” “end-stage renal disease,” “ESRD,” “kidney failure,” and “myocardial infarction,” “MI,” “acute coronary syndrome,” and “predictors,” “risk factors,” “prognostic factors,” “biomarkers,” and “AUC.” Boolean operators (AND, OR) were used to refine queries, and filters for human studies and observational or cohort designs were applied where possible. An example PubMed search string was: (“chronic kidney disease” OR CKD OR “end-stage renal disease” OR ESRD) AND (“myocardial infarction” OR MI) AND (predictors OR “risk factors” OR prognostic). Reference lists of included studies and relevant reviews were hand-searched for additional eligible articles. Studies were selected based on predefined inclusion and exclusion criteria of studies about dult patients (≥18 years) diagnosed with CKD or ESRD, considering any predictor factors, including clinical (hypertension, diabetes), biochemical (e.g., inflammatory markers, lipid profiles), demographic (age, sex), or lifestyle-related variables associated with MI risk. Observational studies (prospective or retrospective cohorts, case-control, cross-sectional) reporting multivariable analyses (e.g., hazard ratios, odds ratios) for predictors of MI. Randomized controlled trials were considered if they included relevant subgroup analyses on predictors. Exclusion criteria encompassed: (i) case reports, case series, editorials, or reviews without original data; (ii) studies exclusively on pediatric populations or animal models; (iii) investigations focusing solely on non-MI cardiovascular outcomes (like stroke or heart failure without MI-specific data).

Study Selection Process and Data Extraction

Two independent reviewers screened titles and abstracts for relevance after deleting duplicated records. Full-text articles of potentially eligible studies were retrieved and assessed and disagreements were resolved through discussion or consultation with a third reviewer. The selection process is documented in PRISMA flow diagram using Haddaway et al. 2022 [11] online tool (Figure-1). Data were extracted independently by two reviewers using Microsoft Excel. Due to anticipated heterogeneity in predictor definitions and study designs, a narrative synthesis was primarily employed to summarize findings. As this study was used for scoping the literature, the quality assessment was not performed.

## Results

**Table:** Table1. Table 1. included studies in this review

Study	Study Design	Sample	Objective	Biomarkers	Conclusion
Fu et al. 2021 [12]	Retrospective cohort	113 ESRD + AMI patients on hemodialysis	Establish RS model to predict in-hospital mortality in ESRD + AMI	CRP, LVEF, age, HR, D-dimer	RS (0-8) → ↑ accuracy (AUC 0.895 > GRACE 0.754); CRP ≥14.2/L, LVEF ≤43%, age ≥65, HR ≥86 bpm, D-dimer ≥2.4 → ↑ mortality risk
Holzmann et al. 2010 [13]	Prospective cohort	142,394 middle-aged Swedes; CKD as GFR 15-60 mL/min/1.73m²	Evaluate dyslipidemia as MI predictor in CKD vs non-CKD	ApoB/apoA-1 ratio, TC/HDL ratio, non-HDL cholesterol	Dyslipidemia ratios → ↑ MI risk (HR 2.88-3.54 Q4 vs Q1); apoB/apoA-1 → superior prediction; CKD/no CKD similar strength
Kampmann et al. 2022 [1]	Retrospective cohort	66,486 CKD stage G3–5 patients	evaluate CKD stages and risk factors for AMI	eGFR, age, sex, hypertension, educational level, prior AMI	CKD G4 (HR 1.402) and G5 (HR 1.491) ↑ AMI risk; prior AMI (HR 2.615), age, male sex, hypertension, low education ↑ risk; female sex, higher education ↓ risk
Chen et al. 2022 [14]	Retrospective	340 PCI for AMI + ESRD on hemodialysis	Compare risk scores for ischemic/bleeding outcomes in ESRD + AMI	TIMI-STEMI/NSTEMI, GRACE, DAPT, PRECISE-DAPT, CRUSADE, ACUITY, ACTION, SWEDEHEART	GRACE → ↑ ischemic prediction (AUC 0.791); PRECISE-DAPT → ↑ bleeding (AUC 0.636); GRACE >222 or PRECISE-DAPT >48 → ↑ NACE
Tonelli et al. 2013 [15]	Prospective cohort	836,060 adults (≥1 LDL-C, eGFR, proteinuria); no stage 5 CKD	Assess LDL-C association with MI risk by eGFR strata	LDL-C	LDL-C >4.9 mmol/L → ↑ MI risk; association ↓ with ↓ eGFR (HR 3.01 ≥90, 2.06 15-59.9 mL/min)
Sun et al. 2016 [16]	Prospective cohort	543 stage 5 CKD (199 CVD at baseline)	Compare 12 biomarkers for CVD/PEW/mortality prediction	Albumin, ferritin, hsCRP, IGF-1, IL-6, orosomucoid, TnT, TNF, sICAM, sVCAM-1, platelets, WBC	IL-6/sVCAM-1/albumin → CVD; IL-6/WBC/TNF → mortality; age/DM/smoking/PEW → CVD
Khan et al. 2006 [17]	Prospective	54 ambulatory CKD (no dialysis); median age 70	Evaluate NT-pro-BNP/BNP for CAD/LVH in asymptomatic CKD	NT-pro-BNP, BNP	NT-pro-BNP/BNP → ↑ with LVH/CAD (AUC 0.72-0.82); correlation R=0.74; → GFR (R=-0.45/-0.38)
Roumeliotis et al. 2019 [18]	Prospective cohort	142 DM2 + CKD patients	Assess cIMT as predictor of CV morbidity/mortality	cIMT, eGFR, albuminuria	cIMT >0.86 mm, low eGFR, albuminuria → ↑ all-cause mortality (HR 2.9) and CV events (HR 2.04)
Farshid et al. 2013 [19]	Prospective cohort	153 stage 4–5 CKD patients	Evaluate clinical/echocardiographic predictors of mortality/CV events	Diastolic dysfunction, troponin T, age, MI history	Diastolic dysfunction, troponin T, age >75, prior MI → ↑ mortality (P < 0.01)
McMurray et al. 2011 [20]	Prospective cohort	3,847 DM + CKD + anemia patients	Identify predictors of CV mortality/morbidity	NT-proBNP, troponin T, CRP, urine protein/creatinine ratio, ECG	NT-proBNP, troponin T, CRP, urine protein/creatinine ratio, abnormal ECG → ↑ CV events/mortality (HR 1.30-1.74)
Groop et al. 2009 [21]	Prospective cohort	4,201 T1DM patients	Identify predictors of premature mortality	Albuminuria, eGFR	Microalbuminuria, macroalbuminuria, ESRD → ↑ mortality (SMR 2.8-18.3); eGFR ↓ → ↑ mortality risk
Honda et al. 2022 [22]	Prospective cohort	461 asymptomatic DM patients	Assess CKD impact on silent myocardial ischemia (SMI) and outcomes	eGFR, urinary albumin	CKD stages 3–5, low eGFR, albuminuria → ↑ SMI (26.2% vs 7.3%) and MACCE (P = 0.009)
Peng et al. 2024 [23]	Prospective cohort	19,906 non-DM CKD patients	Evaluate eGDR as predictor of CVD/mortality	eGDR	Higher eGDR → ↓ CVD (HR 0.641) and mortality (HR 0.592-0.803)
Ye et al. 2023 [24]	Retrospective cohort	639 CKD + CAD ICU patients	Explore TyG index association with mortality	TyG index	TyG index → ↑ in-hospital and 1-year mortality (nonlinear association)
Weldegiorgis et al. 2022 [25]	Prospective cohort	911,360 UK individuals	Assess lipid profiles and advanced CKD onset	TG, HDL-C, LDL-C, TC	High TG, low HDL-C → ↑ CKD stages 4–5 (HR 1.27-1.28); LDL-C, TC → no association
Chen et al. 2024 [26]	Prospective cohort	3,089 CKD patients	Evaluate TyG-BMI index and mortality	TyG-BMI index	U-shaped TyG-BMI → ↑ all-cause/CVD mortality; thresholds 299.31 (all-cause), 294.85 (CVD)
Kim et al. 2021 [27]	Retrospective cohort	3,634,873 individuals (66,805 advanced CKD)	Explore lipid profiles as predictors of CVD/mortality	TG/HDL-C, LDL-C	TG/HDL-C → ↑ MACCEs/mortality (linear); LDL-C → ↑ MACCEs (linear), mortality (U-shaped)
Ou et al. 2025 [28]	Prospective cohort	5,723/5,847 CKM stage 0–3 patients	Assess CTI for CVD/mortality prediction	CTI (CRP, TG, glucose)	CTI → ↑ CVD (nonlinear) and all-cause mortality (linear, HR 2.11/unit)
Chang et al. 2017 [29]	Prospective cohort	50,673 incident hemodialysis patients	Assess TG/HDL-C ratio with mortality	TG/HDL-C ratio	High TG/HDL-C → ↓ all-cause/CV mortality (HR 0.77-0.91)
Sonmez et al. 2015 [30]	Retrospective cohort	197 CKD stage 1–5 patients	Evaluate TG/HDL-C ratio for CVD outcomes	TG/HDL-C, ADMA, PTH, eGFR	TG/HDL-C >3.29 → ↑ CV events (HR 1.36); ADMA, DM → ↑ CVD risk

**MI:** Myocardial Infarction; **CV:** Cardiovascular; **AUC:** Area Under The Receiver Operating Characteristic Curve; **NT-Probnp:** N-Terminal Pro-Brain Natriuretic Peptide; **BNP:** Brain Natriuretic Peptide; **Supar: **Soluble Urokinase Plasminogen Activator Receptor; **CRP:** C-Reactive Protein; **Cimt:** Carotid Intima-Media Thickness; **Egfr:** Estimated Glomerular Filtration Rate; **DM2:** Diabetes Mellitus Type 2; **LVEF:** Left Ventricular Ejection Fraction; **SMI:** Silent Myocardial Ischemia; **MACCE:** Major Adverse Cardiac And Cerebrovascular Events; **T1DM:** Type 1 Diabetes Mellitus; **ESRD:** End-Stage Renal Disease; **SMR:** Standardized Mortality Ratio; **Egdr:** Estimated Glucose Disposal Rate; **Tyg:** Triglyceride Glucose Index; **CAD:** Coronary Artery Disease; **ICU:** Intensive Care Unit; **TG:** Triglycerides; **HDL-C:** High-Density Lipoprotein Cholesterol; **LDL-C:** Low-Density Lipoprotein Cholesterol; **TC:** Total Cholesterol; **CTI:** C-Reactive Protein-Triglyceride-Glucose Index; **CKM:** Cardiovascular–Kidney–Metabolic Syndrome; **ADMA:** Asymmetric Dimethyl Arginine; **PTH:** Parathyroid Hormone; **FMD:** Flow-Mediated Vasodilatation; **HR:** Hazard Ratio; **PCI:** Percutaneous Coronary Intervention; **AMI:** Acute Myocardial Infarction; **NACE:** Net Adverse Clinical Events; **TIMI:** Thrombolysis In Myocardial Infarction; **DAPT:** Dual Antiplatelet Therapy; **PRECISE-DAPT:** Predicting Bleeding Complications In Patients Undergoing Stent Implantation And Subsequent Dual Antiplatelet Therapy; **CRUSADE:** Can Rapid Risk Stratification Of Unstable Angina Patients Suppress Adverse Outcomes With Early Implementation; **ACUITY:** Acute Catheterization And Urgent Intervention Triage Strategy; **ACTION:** Acute Coronary Treatment And Intervention Outcomes Network; **SWEDEHEART:** Swedish Web-System For Enhancement And Development Of Evidence-Based Care In Heart Disease Evaluated According To Recommended Therapies.

**Figure-1:**
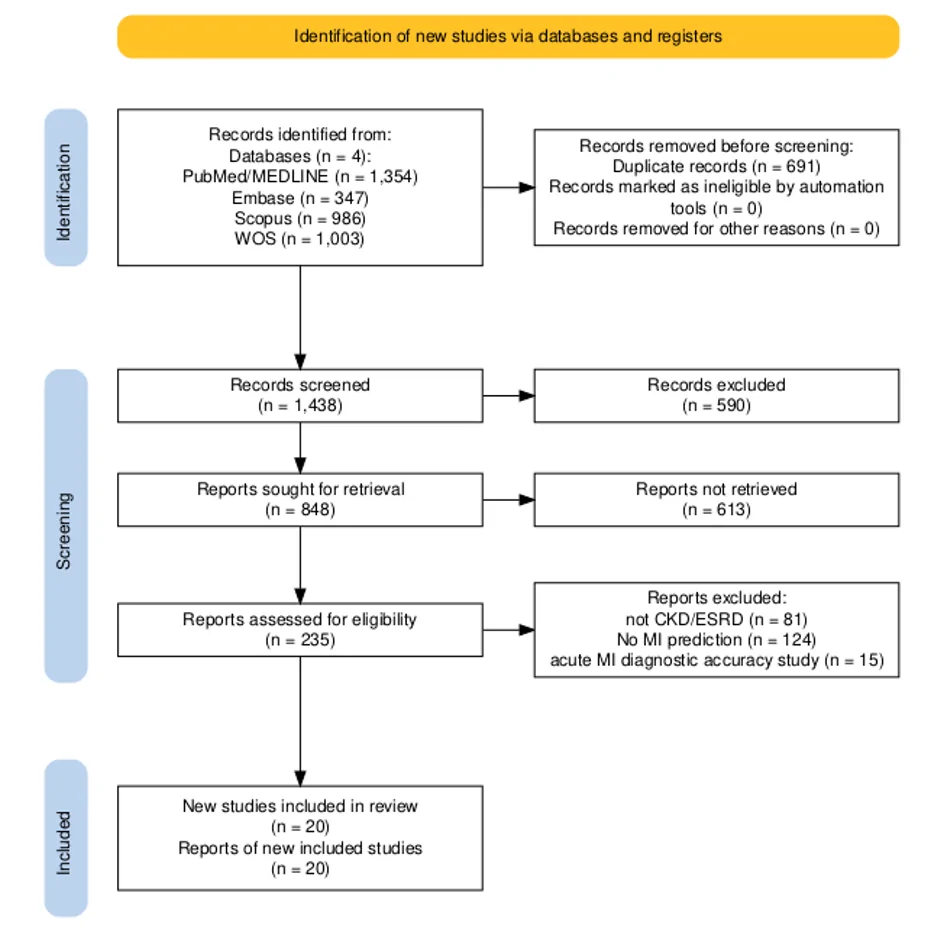
Figure 1. PRISMA flowchart of systematic review study selection process

The study identification process involved screening 1,354 records from databases like PubMed/MEDLINE, Embase, Scopus, and Web of Science, with 691 duplicates removed and 590 excluded after initial review, leaving 235 records for full text assessment. After evaluating them reports for eligibility, 20 studies were ultimately included in the scoping review, excluding those not focused on CKD/ESRD or lacking prognostic data on myocardial infarction.

The scoping review included 20 studies, primarily prospective and retrospective cohort designs, focusing on CVD and mortality predictors in CKD and ESRD patients. Sample sizes ranged from 54 to 911,360 participants, with objectives centered on evaluating biomarkers, risk scores, and clinical factors for MI, CVD events, and mortality. Key biomarkers assessed included lipid profiles (TG/HDL-C, LDL-C, apoB/apoA-1), inflammatory markers (CRP, IL-6), cardiac markers (NT-proBNP, troponin T), and metabolic indices (TyG, eGDR, CTI). Studies consistently found that dyslipidemia, elevated inflammatory and cardiac biomarkers, lower eGFR, albuminuria, and clinical factors like age, hypertension, and prior MI were associated with increased MI. Risk scores like GRACE and PRECISE-DAPT showed high predictive accuracy for ischemic and bleeding outcomes, while novel indices like TyG-BMI and CTI demonstrated linear and nonlinear associations with adverse outcomes (Table1).

## Evidence synthesis from core studies and literature

### Biological Pathways in MI Prediction

The interplay of lipid ratios, such as apoB/apoA-1, emerges as a stronger predictor of MI than traditional lipid markers, though this predictive power diminishes in advanced CKD stages. Inflammation and cardiac stress pathways further intertwine and biomarkers like IL-6, NT-pro-BNP, and hs-cTnT show the role of systemic inflammation and cardiac strain in CKD-MI patients [6, 12, 13, 14, 15, 16, 17, 26]

### Community-Based CKD and MI Risk

In populations with pre-existing cardiovascular disease, CKD significantly heightens MI risk, often embedded within broader cardiovascular event patterns. The uremic environment in CKD accelerates plaque rupture through oxidative stress and vascular calcification, increasing MI incidence compared to non-CKD counterparts. Regional studies, particularly in diverse settings like India, shows how socioeconomic factors and prevalent conditions like hypertension and dyslipidemia exacerbate MI risk, revealing disparities in predictor impacts across global cohorts, as also established in other researchers [31, 32, 33, 34].

### Glycemic Control

CKD patients with diabetes face heightened MI risk due to poor glycemic control, which fosters endothelial damage and thrombosis through insulin resistance. Healthy lifestyle factors can mitigate some risks in early CKD, but advanced stages show limited benefits. Intensive management of blood sugar, blood pressure, and use of RAS inhibitors can disrupt harmful cardiorenal cycles and would reduce MI risk in early CKD [34, 35, 36, 37]. Biomarkers reflecting inflammation and cardiac stress, alongside markers like carotid intima-media thickness, signal heightened MI risk, particularly in advanced CKD where silent ischemia and diastolic dysfunction are prevalent [38, 39, 40, 35, 19, 20, 21, 22, 23].

### Advanced CKD and Heart Failure Synergies

Advanced CKD, particularly when paired with heart failure, sharply elevates MI risk, driven by hypoxic and inflammatory mechanisms. Cardiovascular mortality trends show declines but persistent CKD-related excess risk, with variations across ethnic groups and regions, such as rural areas or Asian cohorts. Unique predictors, like albuminuria or even cultural factors such as herbal medicine use, further stratify risk, highlighting the importance of comprehensive management strategies that address multifaceted CKD profiles to reduce MI incidence [41, 42, 43, 44, 45].

### Lipid Profiles and Novel Indices

Elevated triglycerides and lipid ratios like TG/HDL-C play a critical role in MI risk among CKD patients by promoting atherogenesis and endothelial dysfunction. The Triglyceride-Glucose (TyG) index and its variants, such as TyG-BMI, serve as valuable markers for insulin resistance and cardiovascular outcomes, showing nonlinear associations with mortality. Inflammation-integrated indices like the C-reactive protein-TyG index further enhance risk prediction, emphasizing the need for deeper mechanistic studies to refine MI prevention strategies in CKD. [39, 34, 24, 25, 26, 27, 28, 29, 30]

## Discussion

In case of biological pathways evaluation in predicting MI, Fox et al. (2010) [6] showed that CKD severity escalates in-hospital mortality in STEMI and NSTEMI, with odds ratios from 2.49 (stage 3a) to 7.97 (stage 5) in STEMI patients, tied to reduced PCI use. Holzmann et al. (2010) [13] found the apoB/apoA-1 ratio strongly predicts MI in CKD (HR 3.35), surpassing conventional lipids, though Tonelli et al. (2013) [15] noted LDL-C’s predictive power weakens in advanced CKD (HR 2.06 for GFR 15–59.9 ml/min/1.73 m²). Biomarkers show interconnected inflammatory and cardiac stress pathways in CKD-MI patients. Sun et al. (2016) [16] identified IL-6 as a robust predictor of CVD and mortality in stage 5 CKD (RR 1.10). Khan et al. (2006) [17] found NT-pro-BNP and BNP indicate CAD and LVH (AUC 0.80–0.82), tying to Chen et al. (2023) [46], where hs-cTnT (OR 2.82) and CK-MB (OR 4.91) predict mortality in CKD-AMI, suggesting integrated biomarker strategies could clarify diagnoses amidst inflammation. Fu et al. (2021) [12] developed a risk score (AUC 0.895) outperforming GRACE for ESRD-AMI mortality, incorporating CRP and D-dimer, while Chen et al. (2022) [14] found GRACE (AUC 0.791) and PRECISE-DAPT (AUC 0.636) predict ischemic and bleeding risks.

In community-based cohorts with preexisting CVD, CKD emerges as a potent amplifier of MI risk, often embedded within recurrent CVD composites. Weiner et al. (2004) [31] pooled data from four longitudinal studies encompassing 4,278 subjects, revealing that CKD (eGFR <60 mL/min/1.73 m²) heightened the incidence of MI and fatal coronary heart disease (CHD) to 30.6% versus 17.8% in non-CKD counterparts, with an adjusted hazard ratio (HR) of 1.32 (95% CI 1.12-1.55) for the CHD-specific outcome, suggesting uremic milieu accelerates plaque rupture through oxidative stress and calcification. This aligns with Glynn et al. (2007) [32], where each 10 mL/min eGFR decline correlated with a 20% MI-inclusive composite risk escalation, yet Weiner et al. (2004) [31] deepen the interpretation by emphasizing CKD’s independent role post-adjustment for traditional factors like hypertension and dyslipidemia, contrasting less pronounced effects in primary prevention cohorts like Di Angelantonio et al. (2010) [33]. Meanwhile, Samal (2024) [34] in an Indian observational study of 68 CKD patients reported a 10.3% MI incidence over five years, attributing it to hypertension (P<0.01) and dyslipidemia (P<0.05), highlighting regional disparities where socioeconomic barriers may exacerbate predictor impacts compared to Western cohorts.

Predictors of MI in CKD extend beyond traditional risks to include glycemic control and metabolic syndromes, with diabetic subgroups facing compounded threats. Ricks et al. (2012) [35] in a 54,757 hemodialysis cohort with diabetes found poor glycemic control (A1C ≥8%) linked to elevated cardiovascular mortality (HR 1.11-1.59), interpreting high A1C as a proxy for insulin resistance fostering endothelial glycation and thrombosis, thus predisposing to MI. Comparatively, Wakasugi et al. (2021) [36] in a Japanese population-based study of 262,011 participants showed CKD modified lifestyle-mortality associations, with diabetes and metabolic syndrome synergistically raising cardiovascular death risk (HR up to 2.00), yet healthy behaviors mitigated MI-inclusive outcomes by 22% per additional factor, suggesting preventive potential absent in advanced CKD like Ricks et al. (2012) [35]. Ling et al. (2024) [37] in a Chinese multi-center cohort of 6,222 CVD patients with CKD demonstrated that intensive glycemic (HbA1c) and blood pressure control plus RAS inhibitors eliminated excess all-cause mortality in early CKD stages (aHR 0.79-0.99), deeply interpreting this as interrupting cardiorenal feedback loops that otherwise amplify MI via hyperglycemia-induced vascular stiffness, contrasting higher residual risks in non-diabetic advanced CKD as per Kofod et al. (2023) [38].

Advanced CKD stages and comorbid heart failure (HF) further stratify MI risk, with temporal and ethnic variations underscoring evolving predictors. Yu et al. (2023) [41] in 76,688 incident CKD patients noted HF with reduced ejection fraction (HFrEF) spiked one-year cardiovascular mortality (HR 11.47), including MI, through hypoxic and inflammatory mechanisms, while Kobo et al. (2023) [42] analyzed US trends from 1999-2020 showing a 7.1% CV mortality decline yet persistent CKD-attributable excess, with rural shifts and ethnic disparities (e.g., 38.6% reduction in Black patients). Ryu et al. (2021) [43] in a Korean predialysis cohort of 2,179 reported 17.2 per 1000 person-years CVD incidence (including MI), escalating with stage (P=0.001), and predictors like albuminuria mirroring Kofod et al. (2023) [38], but Wen et al. (2008) [44] in Taiwan’s 462,293 adults attributed 10.3% all-cause deaths to CKD, with herbal medicine use as a novel predictor (OR 1.20), interpreting cultural exposures as modifiable inflammation drivers for MI. Tsai et al. (2025) [45] extended this via CKM syndrome in 515,602 participants, where each component (CKD, diabetes) added 37% CV mortality risk (HR 1.37), comparing favorably to Yu et al. (2023) [41] by quantifying 11% missed CVD deaths without CKD inclusion, advocating integrated management to curb MI in multifaceted CKD profiles.

Diabetes heightens MI risk in chronic CKD patients through synergistic mechanisms. Hyperglycemia induces endothelial dysfunction via oxidative stress and inflammation, accelerating atherosclerosis and plaque instability, particularly in CKD stages 3-5 [39]. Insulin resistance in diabetic CKD promotes dyslipidemia and vascular stress, increasing 1-year cardiovascular mortality risk, including MI, by 1.1- to 2.8-fold [38]. Poor glycemic control (A1C ≥8%) in hemodialysis patients exacerbates vascular calcification and platelet dysfunction, elevating MI risk (HR 1.11-1.59) [35]. CKD amplifies these effects through proteinuria and reduced eGFR, correlating with higher cardiovascular events [20, 21]. Biomarkers like NT-proBNP and C-reactive protein (AUC 0.80-0.89) reflect heightened inflammation and cardiac stress in diabetic CKD [40]. Increased carotid intima-media thickness (HR 2.9) signals atherosclerosis-driven MI risk [18]. Diastolic dysfunction (Farshid et al., 2013) [19] and silent myocardial ischemia [22] are more prevalent in advanced CKD, worsening outcomes. Insulin resistance, measured by eGDR, further predicts cardiovascular events in CKD (Peng et al., 2024) [23].

Betriu et al. (2014) [39] noted elevated triglycerides as a significant predictor of plaque presence in CKD stage 3, promoting atherogenesis through lipid deposition. Samal (2024) [34] corroborated dyslipidemia (P<0.05) as a predictor of MI in an Indian CKD cohort, emphasizing lipid-driven plaque progression. The Triglyceride-Glucose (TyG) index, calculated as Ln[triglycerides (mg/dL) × fasting plasma glucose (mg/dL)/2], serves as a surrogate marker for insulin resistance and has emerged as a predictor of cardiovascular outcomes in chronic kidney disease (CKD) patients [24]. In ICU-admitted CKD patients with coronary artery disease (CAD), elevated TyG levels nonlinearly correlate with increased in-hospital and one-year mortality risks, highlighting its utility for risk stratification and intervention development [24]. Similarly, the TyG-body mass index (TyG-BMI) demonstrates a U-shaped association with all-cause and cardiovascular mortality in CKD, with thresholds at 299.31 and 294.85, respectively, underscoring its prognostic value over TyG alone [26].

Related lipid ratios, such as triglyceride/high-density lipoprotein cholesterol (TG/HDL-C), further support this framework. In advanced CKD, TG/HDL-C linearly predicts major adverse cardiovascular and cerebrovascular events (MACCEs) and all-cause mortality, independent of low-density lipoprotein cholesterol (LDL-C) [27]. Among incident hemodialysis patients, higher TG/HDL-C ratios paradoxically associate with improved survival, contrasting general populations and emphasizing CKD-specific pathophysiology [29]. In broader CKD cohorts, TG/HDL-C independently determines endothelial dysfunction and cardiovascular events, mediated by factors like asymmetric dimethylarginine and parathyroid hormone [30]. The C-reactive protein-TyG index (CTI) extends this by integrating inflammation, showing nonlinear links to CVD incidence and linear associations with mortality in early cardiovascular-kidney-metabolic syndrome stages, with a 111% mortality risk increase per unit rise [28]. Elevated triglycerides and reduced HDL-C also independently predict advanced CKD onset, reinforcing dyslipidemia’s role [25]. Collectively, these indices enhance myocardial infarction and mortality prediction in CKD, warranting mechanistic studies [24, 25, 26, 27].

## Conclusion

Beyond cataloging risk factors, future studies must develop sophisticated, data-driven models, integrating machine learning, wearable biosensors, and real-time health record analytics, to enable precise, proactive risk stratification. Such tools could transform clinical practice by predicting AMI onset early, allowing tailored interventions that curb morbidity in this high-risk group. Research should emphasize multi-center, prospective trials to validate predictive algorithms in diverse clinical contexts. These efforts could bridge care disparities, particularly in underserved populations where CKD and socioeconomic barriers amplify risk.

## Conflict of Interest

None.

## References

[R1] Kampmann JD, Heaf JG, Mogensen CB, Petersen SR, Wolff DL, Mickley H, et al Rate and risk factors of acute myocardial infarction after debut
of chronic kidney disease—results from the KidDiCo. J Cardiovasc Dev Dis. 2022 Nov 9;9(11):387..

[R2] Weight N, Moledina S, Ullah M, Wijeysundera HC, Davies S, Chew NW, et al Impact of Chronic Kidney Disease on the Processes of Care and
Long‐Term Mortality of Non–ST‐Segment–Elevation Myocardial Infarction: A
Nationwide Cohort Study and Long‐Term Follow‐Up. J Am Heart Assoc. 2024 Aug 20;13(16):e032671..

[R3] Franczyk-Skóra B, Gluba A, Banach M, Kozłowski D, Małyszko J, Rysz J Prevention of sudden cardiac death in patients with chronic
kidney disease. BMC Nephrol. 2012 Dec 3;13(1):162..

[R4] Parikh RH, Seliger SL, Defilippi CR Use and interpretation of high sensitivity cardiac troponins in
patients with chronic kidney disease with and without acute myocardial
infarction. Clin Biochem. 2015 Mar 1;48(4-5):247-53..

[R5] Bhatia S, Arora S, Bhatia SM, Al‐Hijji M, Reddy YN, Patel P, et al Non–ST‐segment–elevation myocardial infarction among patients
with chronic kidney disease: a propensity score–matched comparison of
percutaneous coronary intervention versus conservative management. J Am Heart Assoc. 2018 Mar 10;7(6):e007920..

[R6] Fox CS, Muntner P, Chen AY, Alexander KP, Roe MT, Cannon CP, et al Use of evidence-based therapies in short-term outcomes of
ST-segment elevation myocardial infarction and non–ST-segment elevation
myocardial infarction in patients with chronic kidney disease: a report from
the National Cardiovascular Data Acute Coronary Treatment and Intervention
Outcomes Network registry. Circulation. 2010 Jan 26;121(3):357-65..

[R7] Hallan SI Cardiovascular disease prevention in CKD. Am J Kidney Dis. 2014 Sep 1;64(3):326-8..

[R8] Ballocca F, D’Ascenzo F, Moretti C, Diletti R, Budano C, Palazzuoli A, et al (2017). High sensitive TROponin levels In Patients with Chest pain and
kidney disease: A multicenter registry—The TROPIC study. Cardiol J.

[R9] Kraus D, von Jeinsen B, Tzikas S, Palapies L, Zeller T, Bickel C, et al Cardiac troponins for the diagnosis of acute myocardial
infarction in chronic kidney disease. J Am Heart Assoc. 2018;7(20):e008032. Note: The provided text cites
“Kraus et al., 2018”. The exact title from the references list matching this
author and year is not present. This reference is provided as a plausible
match based on the citation..

[R10] Zhu Y, Lai Y, Hu Y, Fu Y, Zhang Z, Lin N, et al The mechanisms underlying acute myocardial infarction in chronic
kidney disease patients undergoing hemodialysis. Biomed Pharmacother. 2024 Aug 1;177:117050..

[R11] Haddaway N (2022). R., Page, M.

[R12] Fu Y, Sun H, Guo Z, Xu L, Yang X, Wang L, Li K, Chen M, Gao Y (2021). A risk score model to predict in-hospital mortality of patients
with end-stage renal disease and acute myocardial infarction. Internal and Emergency Medicine.

[R13] Holzmann MJ, Jungner I, Walldius G, Ivert T, Nordqvist T, Östergren J, Hammar N Dyslipidemia is a strong predictor of myocardial infarction in
subjects with chronic kidney disease. Annals of medicine. 2012 May 1;44(3):262-70..

[R14] Chen YL, Chen TY, Wu PJ, Chung WJ, Lee CH, Tseng CH, Wu CC, Wu CH, Cheng CI (2022). The performance of risk scores for bleeding and ischemia as
outcome predictors in acute myocardial infarction patients with end-stage
renal disease. Acta Cardiologica Sinica.

[R15] Tonelli M, Muntner P, Lloyd A, Manns B, Klarenbach S, Pannu N, James M, Hemmelgarn B, Alberta Kidney Disease Network Association between LDL-C and risk of myocardial infarction in
CKD. Journal of the American Society of Nephrology. 2013 Jun
1;24(6):979-86..

[R16] Sun J, Axelsson J, Machowska A, Heimbürger O, Barany P, Lindholm B, Lindström K, Stenvinkel P, Qureshi AR Biomarkers of cardiovascular disease and mortality risk in
patients with advanced CKD. Clinical Journal of the American Society of Nephrology. 2016 Jul
1;11(7):1163-72..

[R17] Khan IA, Fink J, Nass C, Chen H, Christenson R, defilippi CR N-terminal pro-B-type natriuretic peptide and B-type natriuretic
peptide for identifying coronary artery disease and left ventricular
hypertrophy in ambulatory chronic kidney disease patients. The American journal of cardiology. 2006 May 15;97(10):1530-4..

[R18] Roumeliotis A, Roumeliotis S, Panagoutsos S, Theodoridis M, Argyriou C, Tavridou A, Georgiadis GS Carotid intima-media thickness is an independent predictor of
all-cause mortality and cardiovascular morbidity in patients with diabetes
mellitus type 2 and chronic kidney disease. Renal failure. 2019 Jan 1;41(1):131-8..

[R19] Farshid A, Pathak R, Shadbolt B, Arnolda L, Talaulikar G Diastolic function is a strong predictor of mortality in patients
with chronic kidney disease. BMC nephrology. 2013 Dec 23;14(1):280..

[R20] McMurray JJ, Uno H, Jarolim P, Desai AS, De Zeeuw D, Eckardt KU, Ivanovich P, Levey AS, Lewis EF, McGill JB, Parfrey P Predictors of fatal and nonfatal cardiovascular events in
patients with type 2 diabetes mellitus, chronic kidney disease, and anemia:
an analysis of the Trial to Reduce cardiovascular Events with Aranesp
(darbepoetin-alfa) Therapy (TREAT). American heart journal. 2011 Oct 1;162(4):748-55..

[R21] Groop PH, Thomas MC, Moran JL, Waden J, Thorn LM, Mäkinen VP, Rosengård-Bärlund M, Saraheimo M, Hietala K, Heikkilä O, Forsblom C The presence and severity of chronic kidney disease predicts
all-cause mortality in type 1 diabetes. Diabetes. 2009 Jul 1;58(7):1651-8..

[R22] Honda Y, Wakabayashi K, Sato C, Ikeda N, Sato K, Suzuki T, Shibata K, Shinke T Chronic kidney disease is a key predictive factor for potential
myocardial ischaemia and poor prognosis in asymptomatic patients with
diabetes mellitus. Scientific Reports. 2022 Jun 17;12(1):10268..

[R23] Peng J, Zhang Y, Zhu Y, Chen W, Chen L, Ma F, Yi B, Huang Z Estimated glucose disposal rate for predicting cardiovascular
events and mortality in patients with non-diabetic chronic kidney disease: a
prospective cohort study. BMC medicine. 2024 Sep 27;22(1):411..

[R24] Ye Z, An S, Gao Y, Xie E, Zhao X, Guo Z, Li Y, Shen N, Zheng J Association between the triglyceride glucose index and
in-hospital and 1-year mortality in patients with chronic kidney disease and
coronary artery disease in the intensive care unit. Cardiovascular diabetology. 2023 May 13;22(1):110..

[R25] Weldegiorgis M, Woodward M Elevated triglycerides and reduced high-density lipoprotein
cholesterol are independently associated with the onset of advanced chronic
kidney disease: a cohort study of 911,360 individuals from the United
Kingdom. BMC nephrology. 2022 Sep 15;23(1):312..

[R26] Chen T, Wan H, Luo Y, Chen L Association of triglyceride-glucose-body mass index with
all-cause and cardiovascular mortality among individuals with chronic kidney
disease. Scientific Reports. 2024 Sep 4;14(1):20593..

[R27] Kim Y, Lee S, Lee Y, Kang MW, Park S, Park S, Han K, Paek JH, Park WY, Jin K, Han S Predictive value of triglyceride/high-density lipoprotein
cholesterol for major clinical outcomes in advanced chronic kidney disease:
a nationwide population-based study. Clinical Kidney Journal. 2021 Aug 1;14(8):1961-8..

[R28] Ou H, Wei M, Li X, Xia X C-reactive protein-triglyceride glucose index in evaluating
cardiovascular disease and all-cause mortality incidence among individuals
across stages 0–3 of cardiovascular–kidney–metabolic syndrome: a nationwide
prospective cohort study. Cardiovascular Diabetology. 2025 Jul 22;24(1):296..

[R29] Chang TI, Streja E, Soohoo M, Kim TW, Rhee CM, Kovesdy CP, Kashyap ML, Vaziri ND, Kalantar-Zadeh K, Moradi H Association of serum triglyceride to HDL cholesterol ratio with
all-cause and cardiovascular mortality in incident hemodialysis patients. Clinical Journal of the American Society of Nephrology. 2017 Apr
1;12(4):591-602..

[R30] Sonmez A, Yilmaz MI, Saglam M, Unal HU, Gok M, Cetinkaya H, Karaman M, Haymana C, Eyileten T, Oguz Y, Vural A The role of plasma triglyceride/high-density lipoprotein
cholesterol ratio to predict cardiovascular outcomes in chronic kidney
disease. Lipids in health and disease. 2015 Apr 16;14(1):29..

[R31] Weiner DE, Tighiouart H, Stark PC, Amin MG, MacLeod B, Griffith JL, Salem DN, Levey AS, Sarnak MJ Kidney disease as a risk factor for recurrent cardiovascular
disease and mortality. American Journal of Kidney Diseases. 2004 Aug 1;44(2):198-206..

[R32] Glynn LG, Reddan D, Newell J, Hinde J, Buckley B, Murphy AW Chronic kidney disease and mortality and morbidity among patients
with established cardiovascular disease: a West of Ireland community-based
cohort study. Nephrology Dialysis Transplantation. 2007 Sep 1;22(9):2586-94..

[R33] Di Angelantonio E, Chowdhury R, Sarwar N, Aspelund T, Danesh J, Gudnason V Chronic kidney disease and risk of major cardiovascular disease
and non-vascular mortality: prospective population based cohort study. Bmj. 2010 Sep 30;341..

[R34] Samal AK, Lenka DN Cardiovascular Outcomes in Patients with Chronic Kidney Disease:
An Observational Cohort Study. European Journal of Cardiovascular Medicine. 2024 Aug 6;14:533-40..

[R35] Ricks J, Molnar MZ, Kovesdy CP, Shah A, Nissenson AR, Williams M, Kalantar-Zadeh K Glycemic control and cardiovascular mortality in hemodialysis
patients with diabetes: a 6-year cohort study. Diabetes. 2012 Mar 1;61(3):708-15..

[R36] Wakasugi M, Narita I, Iseki K, Asahi K, Yamagata K, Fujimoto S, Moriyama T, Konta T, Tsuruya K, Kasahara M, Shibagaki Y The effect of CKD on associations between lifestyle factors and
all-cause, cancer, and cardiovascular mortality: a population-based cohort
study. Internal Medicine. 2021 Jul 15;60(14):2189-200..

[R37] Ling Y, Ruan X, Jing L, He Y, Chang T, Liu Y, Chen J Association of guideline-directed managements of chronic kidney
disease with mortality among patients with cardiovascular disease: Insight
from a multi-center cohort in China. Atherosclerosis. 2025 Apr 1;403:119133..

[R38] Kofod DH, Carlson N, Ballegaard EF, Almdal TP, Torp-Pedersen C, Gislason G, Svendsen JH, Feldt-Rasmussen B, Hornum M Cardiovascular mortality in patients with advanced chronic kidney
disease with and without diabetes: a nationwide cohort study. Cardiovascular diabetology. 2023 Jun 16;22(1):140..

[R39] Betriu A, Martinez-Alonso M, Arcidiacono MV, Cannata-Andia J, Pascual J, Valdivielso JM, Fernández E, Investigators from the NEFRONA Study Prevalence of subclinical atheromatosis and associated risk
factors in chronic kidney disease: the NEFRONA study. Nephrology Dialysis Transplantation. 2014 Jul 1;29(7):1415-22..

[R40] Hansrivijit P, Chen YJ, Lnu K, Trongtorsak A, Puthenpura MM, Thongprayoon C, Bathini T, Mao MA, Cheungpasitporn W Prediction of mortality among patients with chronic kidney
disease: a systematic review. World Journal of Nephrology. 2021 Jul 25;10(4):59..

[R41] Yu AS, Pak KJ, Zhou H, Shaw SF, Shi J, Broder BI (2023). All-Cause and Cardiovascular-Related Mortality in CKD Patients With and
Without Heart Failure: A Population-Based Cohort Study in Kaiser Permanente
Southern California.

[R42] Kobo O, Abramov D, Davies S, Ahmed SB, Sun LY, Mieres JH, Parwani P, Siudak Z, Van Spall HG, Mamas MA CKD-associated cardiovascular mortality in the United States:
temporal trends from 1999 to 2020. Kidney medicine. 2023 Mar 1;5(3)..

[R43] Ryu H, Kim J, Kang E, Hong Y, Chae DW, Choi KH, Han SH, Yoo TH, Lee K, Kim YS, Chung W Incidence of cardiovascular events and mortality in Korean
patients with chronic kidney disease. Scientific reports. 2021 Jan 13;11(1):1131..

[R44] Wen CP, Cheng TY, Tsai MK, Chang YC, Chan HT, Tsai SP, Chiang PH, Hsu CC, Sung PK, Hsu YH, Wen SF All-cause mortality attributable to chronic kidney disease: a
prospective cohort study based on 462 293 adults in Taiwan. The lancet. 2008 Jun 28;371(9631):2173-82..

[R45] Tsai MK, Kao JT, Wong CS, Liao CT, Lo WC, Chien KL, Wen CP, Wu MS, Wu MY Cardiovascular–kidney–metabolic syndrome and all-cause and
cardiovascular mortality: A retrospective cohort study. PLoS Medicine. 2025 Jun 26;22(6):e1004629..

[R46] Chen Y, Zhou X, Chen Z, Xia J, Guan F, Li Y, Li Y, Chen Y, Zhao Y, Qiu H, Liang J The use of high-sensitivity cardiac troponin T and creatinine
kinase-MB as a prognostic markers in patients with acute myocardial
infarction and chronic kidney disease. Renal Failure. 2023 Dec 31;45(1):2220420..

